# Blood Purification in Severe and Critical COVID-19 Patients: A Case Series of 5 Patients

**DOI:** 10.3389/fpubh.2021.741125

**Published:** 2021-11-17

**Authors:** Hongjun Chen, Leisheng Zhang, Wei Zhang, Lili Liu, Zhihua Dai, Miao Chen, Donghang Zhang

**Affiliations:** ^1^Department of Intensive Care Unit, Affiliated Hospital of Zunyi Medical University, Zunyi, China; ^2^Stem Cell Bank, Guizhou Health-Biotech Biotechnology Co., Ltd., Guiyang, China; ^3^Precision Medicine Division, Health-Biotech (Tianjin) Stem Cell Research Institute Co., Ltd., Tianjin, China; ^4^Department of Neurosurgery, The First Affiliated Hospital, Shandong First Medical University, Jinan, China; ^5^Jiangxi Research Center of Stem Cell Engineering, Jiangxi Health-Biotech Stem Cell Technology Co., Ltd., Shangrao, China; ^6^Department of Cerebrovascular Surgery, Affiliated Hospital of Zunyi Medical University, Zunyi, China; ^7^Department of Anesthesiology, West China Hospital, Sichuan University, Chengdu, China

**Keywords:** COVID-19, blood purification, inflammatory cytokine storm, hemofiltration, hemodiafiltration

## Abstract

**Objective:** The ongoing coronavirus disease 2019 (COVID-19) caused by the severe acute respiratory syndrome coronavirus 2 (SARS-CoV-2) has led to a worldwide pandemic. Currently, supportive care measures remain the standard of care for severe and critical COVID-19 patients, such as ventilation oxygenation, fluid management and blood purification. In this study, we aimed to evaluate the effects of early blood purification therapy upon severe and/or critical COVID-19 patients.

**Patients and Methods:** From January 31, 2020 to March 1, 2020, a total 5 patients with COVID-19 (3 critical type cases and 2 severe type cases) received early blood purification treatment in the intensive care unit (ICU) of Affiliated Hospital of Zunyi Medical University. Clinical indexes, including oxygen concentration, blood gas analysis, oxygenation index, and laboratory test as well as disease scores were recorded and analyzed before and after the treatment with blood purification.

**Results:** Among the 5 patients, 4 were males ranging from 35 to 80 year old (Mean age = 63 ± 17.87). All cases with characteristics of OI <300 mm Hg, decline in lymphocyte (LYMPH)%, boost in lactate dehydrogenase (LDH), troponin T (TNT), B-type brain natriuretic peptide (BNP), interleukin-6 (IL-6) and interferon-alpha (IFN-a), three with high flow nasal cannula (HFNC), two with non-invasive ventilation (NIV) and acute kidney injury (AKI), and one with shock and IV. Blood purification therapy significantly decreased the serum levels of inflammatory cytokine, ameliorated the concomitant symptoms and complications. Finally, one case was discharged from the hospital, 4 cases were transferred to the general ward, and all the 5 cases survived.

**Conclusion:** Continuous blood purification therapy held promising prospects for alleviating the deteriorative progression of severe and critical types of COVID-19 in the early stage, together with ameliorating the accumulation of inflammatory cytokine and the concomitant symptoms and complications by efficacious immunoadsorption.

**Trial Registration:** www.chictr.org.cn, Identifier (ChiCTR2000031930).

## Introduction

The corona virus disease 2019 (COVID-19) pandemic, caused by severe acute respiratory syndrome coronavirus 2 (SARS-CoV-2), emerged in late 2019 and has infected tens of millions of people worldwide (1, 2). Patients with COVID-19 manifested various symptoms, including fever, dry cough, headache, pneumothorax, fatigue, nausea, low oxygen saturation, infiltrating ground-glass opacity in lungs, shock, oliguria, and the multiple organ dysfunction syndrome (MODS) (2–5). Severe or critical COVID-19 patients are usually characterized by severe complications, such as pneumonia-associated acute lung injury and acute respiratory distress syndrome (ALI/ARDS), liver injury, cardiac failure and acute kidney injury (AKI), which collectively resulted in poor prognosis and high mortality (3, 5, 6). Inflammatory cytokine storm, characterized by an uncontrolled release of inflammatory mediators, represents a common feature and is suggested to be an important predicter of disease severity for severe or critical COVID-19 patients (7). Therefore, inflammatory cytokines might be a major therapeutic target for severe or critical COVID-19 patients.

To date, comprehensive treatments have been established and optimized for conquering the COVID-19 associated clinical presentations and the concomitant cytokine storm and multiple complications, including antiviral drugs, antibiotics, vaccines, corticosteroids, immunotherapeutics, mesenchymal stem cell- or natural killer cell-based cytotherapy, and supportive therapeutics (8–11). Of them, continuous blood purification administration by continuous venovenous hemofiltration/hemodiafiltration (CVVH/CVVHDF) has been demonstrated with preferable effects in rapid elimination of abnormally elevated proinflammatory cytokines in SARS-CoV-infected patients in 2003, which thus indicates an off-the-shelf strategy for COVID-19 infection (12). However, the systematic and detailed information upon patients with COVID-19 infection and the relevant comorbidities is still far from satisfaction (13). Several studies have reported that blood purification can improve the clinical outcomes of severe or critical COVID-19 patients by decreasing the excess levels of inflammatory cytokines from blood (12, 14–16).

For the purpose, in this study, we enrolled five cases with severe or critical type of COVID-19 infection from the ICU in Affiliated Hospital of Zunyi Medical University and conducted blood purification treatment with the consent of the ethical committee and patients or family members from January 31, 2020 to March 1, 2020. In details, in the beginning of hospitalization stage, the administration of the 5 cases (2 with severe type and 3 with critical type) with coronavirus infection-induced pneumonia was based on the recommended treatment options of the fifth version of diagnosis and treatment scheme for novel coronavirus infection-induced pneumonia by the National Heath Commission of the People's Republic of China. After that, considering the none improvement in oxygenation index (OI) or bilateral lung exudation, the five patients received blood purification treatment according to the multiple disciplinary team (MDT) and the consent of the patients or the family members in Affiliated Hospital of Zunyi Medical University (ethical approval No.: KLL-2020-013) as well as the internationally recognized Declaration of Helsinki. Notably, the severe clinical presentations and rock-ribbed cytokine storm caused by proinflammatory factor in peripheral blood as well as the multiple comorbidities were collectively and efficiently rescued to normal without death. Overall, our findings supported the recommendation for the involvement of blood purification treatment for the comprehensive management of COVID-19 patients.

## Case Presentation

### Participants

The five cases with COVID-19 infection, including two with severe type and three with critical type, were definitely confirmed by nucleic acid test (NAT) of SARS-CoV-2 and turned to the intensive care unit (ICU) of Affiliated Hospital of Zunyi Medical University according to the fifth version of diagnosis and treatment scheme for novel coronavirus infection-induced pneumonia by the National Heath Commission of the People's Republic of China. In details, the patients with severe type of COVID-19 infection should satisfy the following criteria including respiratory distress (RR ≥ 30 per min), Pulse oxygen saturation at rest ≤ 93%, or Partial arterial oxygen (PaO_2_)/ Oxygen concentration ratio ≤ 300 mmHg. As to those with critical type should satisfy the following criteria including respiratory failure and mechanical ventilation, shock, or ICU care for other organ failure. The age of these five patients raged from 35 to 80 year old (63 ± 17.87), of which four were male and one was female (Table 1). Several comorbidities existed in three patients, among them two were diagnosed with diabetes mellitus, and one was with coronary heart disease (CAD), malignant tumor, and chronic obstructive pulmonary disease (COPD) (Table 1). All cases manifested typical symptoms of COVID-19, including cough, fever, headache, fatigued and nausea (Table 1). PiO2/FiO2 ratio in all 5 cases was lower than 300 mmHg and flow oxygen uptake (FNC) was high in three cases. Two cases were performed with non-invasive mechanical ventilation (NIV) and one case with invasive mechanical ventilation (IV). In details, on the one hand, patients with severe COVID-19 infection received oxygen therapy via a nasal catheter or mask, and respiratory distress and/or hypoxemia was assessed in a timely manner; on the other hand, patients received high flow nasal catheter oxygen therapy or non-invasive mechanical ventilation (inhaled oxygen concentration should be as low as 60%) when respiratory distress and/or hypoxemia couldn't be alleviated after standard oxygen therapy. Shock occurred in one patient and pneumothorax occurred in two patients (Table 1). All cases showed high levels of lactate dehydrogenase (LDH), myoglobin (MYO) and B-type brain natriuretic peptide (BNP). Two cases developed acute kidney injury (AKI) (Table 1). The major medication included antiviral, antibiotics, immune globulin, thymalfasin, and hormone (Table 1). Only the 75 year old patients with pseudomonas maltophilia infection identified by microculture of sputum also received continuous 0.3–0.5 ug/kg/h of the positive inotropic drug norepinephrine administration via intravenous pumping during hospitalization. As to the statistical analyses, all the data were analyzed by using the Prism v6.0 software (GraphPad, USA) as we previously reported (17–21). All the data were shown as Mean ± SEM (*N* = 5 individuals). ^*^, *P* < 0.05; ^**^, *P* < 0.01; NS, not significant.

**Table 1:** Demographic data and clinical features.

**Items**	**Case number (%)**
Gender (male, *n*%)	4 (80.00)
Age ( ± s) years	(63 ± 17.87)
**Complication**	
Diabetes mellitus	2 (40.00)
COPD	1 (20.00)
Coronary heart disease (CHD)	1 (20.00)
Malignancy	1 (20.00)
**Symptom**	
Fever	3 (60.00)
Cough	2 (40.00)
Fatigue	3 (60.00)
Headache	1 (20.00)
Naupathia	2 (40.00)
Emesis	2 (40.00)
**Complication**	
Myocardial damage	5 (100.00)
Acute kidney injury (AKI)	2 (40.00)
Acute liver injury (ALI)	4 (80.00)
Pneumothorax	1 (20.00)
**Respiratory support**	
Invasive ventilation (IV)	1 (20.00)
Non-invasive ventilation (NIV)	2 (40.00)
High flow oxygen therapy (HFNC)	3 (60.00)
**Remedy**	
Antiviral	5 (100.00)
Antibiotics	5 (100.00)
Immune globulin	1 (20.00)
Thymalfasin	5 (100.00)
Hormone	5 (100.00)
Blood purification	5 (100.00)

*IV, invasive ventilation; NIV, non-invasive ventilation; HFNC, high flow oxygen therapy; AKI, acute liver injury*.

### Blood Purification Administration

All patients received blood purification treatment immediately after admitting into ICU according to the multidisciplinary consultation (MDT) and the internationally recognized Declaration of Helsinki as well as the consent of the patients or family members in Affiliated Hospital of Zunyi Medical University (ethical approval No.: KLL-2020-013). As recommended by the aforementioned guidelines, RRT should be initiated immediately after respiratory support therapy if oxygenation didn't improve, or if respiratory parameters increased with lung imaging infiltration and shadow increased within 6 h. During the hospitalization period (7–60 days), the continuous veno-venous hemofiltration/ hemodiafiltration (CVVH/CVVHDF) was performed for 8–16 h per filter using the M100/Oxiris, heparin or citric acid anticoagulant after establishing the femoral vein single-tube double-lumen access. In details, we took advantage of the Prismaflex (Jinbao, China) with the M100/OXIRIS filter (Baxter, USA) and the commercial dialysis (Qingshanlikang, China) and replacement fluid (Chengdu, Qingshanlikang, China) for CRRT, and the pre-diluted mode of CVVH/CVVHDF was conducted during the treatment. During RRT, the setting of blood flow velocity was 150 mL/min, the prescribed dose and ultrafiltration were 25~30 ml/kg.h and 50~200 ml/h, respectively. Blood gas analysis was conducted and the parameters were adjusted every 2 to 4 h. The termination of CRRT was according to the concentration of inflammatory factors after blood purification, respiratory support intensity, chest CT exudation and urine volume. Additionally, 4% Citric acid hydrochloric acid as well as sodium bicarbonate injection (Huiyinbi, China) and 0.9% sodium chloride (Guizhou Kelun, China) was used for *in vitro* local anticoagulation. For instance, we took advantage of Oxiris if the cases with severe CSS and high respiratory support parameters, while CVVHDF with superiorities in maintaining even running as well as convenience in changing PRISMAFLEX into CVVH, which was unrealizable for CVVH changing into CVVHDF instead. All the CRRT parameters including the blood flow rate, the dialysate rate, the fluid removal rate, and the anticoagulation regimen were set up according to the 2012 version of KDIGO guidelines and the circumstances of each patient rather than the uniform parameters. For example, the blood flow and outflow volume were set as 100–200 ml/min and 20–25 ml/kg/h, respectively. The launch and termination of the following filter of blood purification therapy after the first one was based on the vital signs, respiratory status, oxygenation index, secreted cytokines and lung shadow absorption in imaging of the patients. No adverse effects, such as bleeding, oozing, allergy or shock were observed in the participants during the process of blood purification treatment. Additionally, results of the blood samples were collected and recorded before and after blood purification administration or at six in the morning if in the interval.

## Results

**Figure 1:**
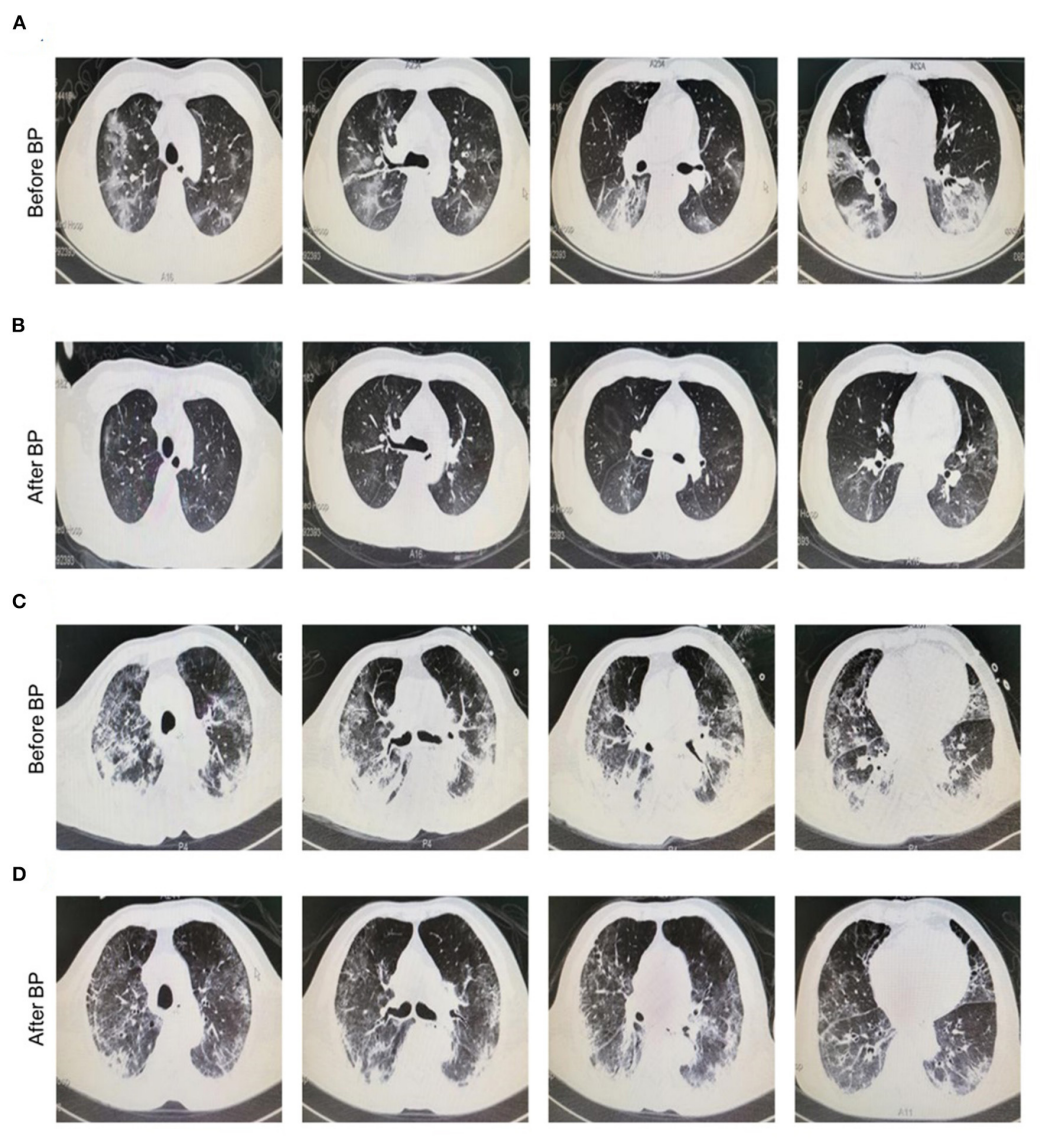
Chest radiographs of COVID-19 patients before and after blood purification. **(A,B)** Representative chest CT manifestation of a 35 year old male patient with severe COVID-19 infection before **(A)** and after **(B)** blood purification treatment. This patient was diagnosed with dyspnea (FiO2, 41%, SpO2, 90%) and received HFNC and blood purification via CVVH/CVVHDF for one time. Finally, he was discharged from hospital with negative result of nuclei acid test (NAT). The typical ground-glass lesion as well as the inflammatory exudate in the lung of the case with severe COVID-19 infection before and after blood purification indicated the favorable prognosis. **(C,D)** Representative chest CT manifestation of a 75 year old male patient with critical COVID-19 infection before **(C)** and after **(D)** blood purification treatment. The lung of the patient with infiltrating ground-glass opacity in imaging before blood purification was significantly alleviated after treatment. The patient was diagnosed with ARDS, MODS, COPD and had comorbidities of type II diabetes and CHD. Before and after blood purification, the patient was positive and negative for NAT for SARS-CoV-2 in pharyngeal swabs, nasal swabs, stools and saliva, and turned negative after treatment, respectively.

**Figure 2:**
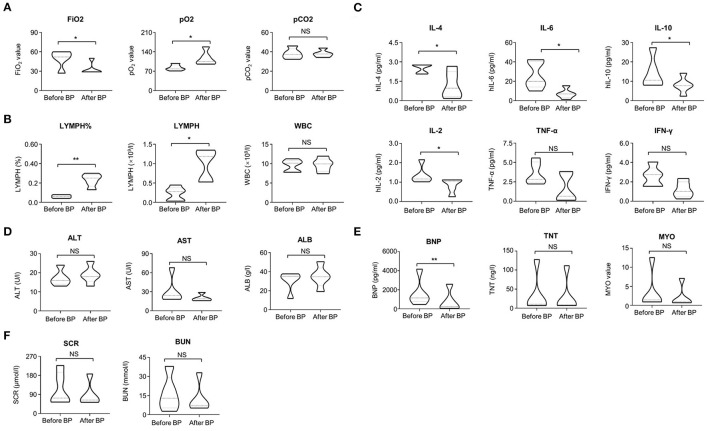
The variations of clinical parameters before and after blood purification. **(A)** The variations of FiO_2_ value, PO_2_ value, PCO_2_ value in the patients before and after blood purification treatment. **(B)** The variations of LYMPH (%), LYMPH and WBC (10^9^/L) in the peripheral blood of the patients before and after blood purification treatment. **(C)** The variations of proinflammatory cytokines (IL-2, IL-4, IL-6, IL-10, TNF-α, IFN-γ) in the peripheral blood of the patients before and after blood purification treatment. **(D)** The variations of AST, ALB, and ALT in the patients before and after blood purification treatment. **(E)** The variations of BNP, TNT, MYO in the patients before and after blood purification treatment. **(F)** The variations of SCR and BUN in the patients before and after blood purification treatment. The data were shown as violin diagrams. All the data were shown as Mean ± SEM (*N* = 5 individuals). *P < 0.05; **P < 0.01; NS, not significant”.

The clinical symptoms of all cases were significantly improved after treatment with continuous blood purification. Of note, the typical ground-glass lesion and the inflammatory exudation in both lungs of one case was largely relieved by blood purification administration for one time (Figures 1A,B). The imaging signs were also partly improved in the other 4 cases (Figures 1C,D). The fraction of inspiration O2 (FiO2) and partial pressure of oxygen (pO2) were improved after blood purification treatment (Figure 2A, Table 2, Supplementary Figure 1A). The declined contents of lymphocytes in peripheral blood were elevated to normal level, whereas no significant change was observed in white blood cells (WBC) (Figure 2B, Table 2, Supplementary Figure 1B). Furthermore, the levels of proinflammatory cytokines, including IL-2, IL-4, IL-6, IL-10 were significantly decreased after blood purification treatment (Figure 2C, Table 2, Supplementary Figure 1C).

**Table 2:** Laboratory indexes before and after blood purification.

**Items**	**Reference value**	**Prior treatment**	**Post-treatment**	***P*-value**
WBC ( × 10^9^/L)	4~10	9.50 ± 1.56	9.69 ± 1.73	0.860
LYMPH%	0.20~0.50	0.08 ± 0.04	0.22 ± 0.08	0.006
HGB (g/L)	130~175	107.00 ± 20.55	103.40 ± 12.30	0.745
PLT ( × 10^9^/L)	100~300	222.20 ± 82.71	237.40 ± 51.94	0.737
ALT (U/L)	9~50	17.20 ± 4.55	19.00 ± 4.69	0.555
SCR (umol/L)	41~109	107.20 ± 62.45	69.80 ± 16.18	0.231
LDH (U/L)	140~271	415.20 ± 119.61	201.80 ± 40.35	0.005
CRP (mg/L)	0.068~8.2	78.32 ± 34.23	11.22 ± 9.14	0.003
FiO2 (%)		49.80 ± 12.66	31.80 ± 4.92	0.016
OI (mmHg)	460~530	191.99 ± 81.13	355.72 ± 131.92	0.05
P (A-a) O2 (mmHg)	25~75	161.16 ± 62.19	66.02 ± 32.22	0.016
BNP (pg/ml)	<125	1643.0 ± 411.3	759.9 ± 287.5	0.006
IL-6 (pg/ml)	1.18~5.30	45.29 ± 33.73	7.21 ± 7.12	0.038
IL-2 (pg/ml)	0.08~5.71	1.36 ± 0.46	0.85 ± 0.38	0.048
IL-10 (pg/ml)	0.19~4.91	14.06 ± 8.09	4.81 ± 1.96	0.038
INF-α (pg/ml)	0.10~2.31	3.26 ± 1.90	1.99 ± 1.75	0.303
TNT (ng/L)	<14	48.86 ± 43.83	13.23 ± 7.45	0.111
APACHE II (score)		15.80 ± 3.49	6.40 ± 0.89	0.001
SOFA (score)		7.80 ± 1.64	3.00 ± 1.25	0.001
PSI (score)		107.00 ± 32.90	29.00 ± 21.04	0.001

*LDH, lactate dehydrogenase; FiO2, fraction of inspiration O2; P(A-a)O2, alveolar gas-arterial blood nitrogen partial pressure difference; OI, oxygenation index; BNP, Type B brain natriuretic peptide; TNT, hypersensitive troponin T; APACHE II, acute physiology and chronic health evaluation scoring system; SOFA, sequential organ failure assessment; PSI, pneumonia severity index*.

Furthermore, the effects of blood purification upon the function of vital organs in severe and critical COVID-19 patients were also evaluated by determining the multifaceted parameters before and after CVVH/CVVHDF. Although a declined trends existed, the change of expression levels of aspartate aminotransferase (AST), albumin (ALB) and alanine transaminase (ALT) showed no statistical differences (*P* > 0.05) (Figure 2D, Table 2, Supplementary Figure 2A). Also, the expression levels of serum creatinine (SCR) and blood urea nitrogen (BUN) were not changed during blood purification (Figure 2F, Table 2, Supplementary Figure 2B). However, the abnormity of brain natriuretic peptide (BNP), but not the troponin T (TNT) and myocardin (MYO) was improved after blood purification treatment (Figure 2E, Table 2, Supplementary Figure 2C). We calculated the multifaceted disease scores (e.g., APACHE II, SOFA, PSI) and found a significant decrease after blood purification treatment (Table 2). Collectively, these results together with the sharp decrease in multifaceted disease scores (e.g., APACHE II, SOFA, PSI; *P* < 0.0001) indicated the partial remission in MODS and the multiple comorbidities (Table 2). None of cases suffered from complications of blood purification including transient hypotension, puncture site bleeding, hematoma, peri-catheter thrombosis, air embolism and infection.

## Discussion

State-of-the-art renewal has revealed the multidimensional characteristics in epidemiology, clinical manifestation and pathogenesis of COVID-19 infection, which collectively facilitate and accelerate the fundamental and clinical studies upon comprehensive treatment (9, 22–24). Cytokines and endotoxins have been correlated with multiple complications such as acute kidney injury (AKI) and septic shock, which can be effectively removed by blood purification treatment (25). According to the diagnosis and treatment scheme, the blood purification treatment was conducted for all patients with severe or critical COVID-19 infection. Generally, all inpatients with favorable prognosis together with remission in clinical indicators such as hemogram, pulmonary ventilation, inflammatory cytokine secretion and disease score. It is noteworthy that early blood purification administration was beneficial to reduce acute lung injury and acute respiratory distress syndrome (ALI/ARDS), improve respiratory condition, facilitate lung regeneration and repair, and finally ameliorate the outcome of patients with severe and critical COVID-19 infection.

Generally, blood purification-based renal replacement therapy (RRT) can be divided into various subtypes, including continuous renal replacement therapy (CRRT), intermittent hemodialysis (IHD), sustained low efficiency dialysis (SLED) and peritoneal dialysis (PD). Differ from other types of RRT, CRRT including CVVH, continuous veno-venous hemodiafiltration (CVVHDF), continuous veno-venous hemodialysis (CVVHD) and slow continuous ultrafiltration (SCUF) has superiority in accurately controlling the solute and volume in critically ill patients. Due to the variations in removing solute and water, the choice of CRRT should be determined based on the degree of convection, dispersion, substitution fluid and dialysis solution (Supplementary Table 1). In particular, CVVHDF possesses the advantages of CVVH and CVVHD and thus the good scavenging ability for small molecules and macromolecules. Taken together, considering the advantages and limitations of CRRT as well as the high level of proinflammatory factors in patients with severe or critical COVID-19 infection, we finally conducted the application of blood purification-based CVVH/CVVHDF mode. Notably, with the aid of the Prismaflex (Jinbao, China)- and the M100/OXIRIS filter (Baxter, USA)-based blood purification, the patients with severe or critical type of COVID-19 infection were recovered from the cytokine storm syndrome, the typical ground-glass lesion and inflammatory exudate in the lung as well as the long-lasting sepsis and SARS-CoV-2 nucleic acid positive.

Patients with severe or critical COVID-19 infection revealed typical symptoms (e.g., fever, cough, fatigue), lungs with infiltrating ground-glass opacity in imaging, together with multiple complications (e.g., acute kidney injury, liver injury, cardiac failure, ALI/ARDS), and thus the resultant poor prognosis. Despite no uniform conclusion was drawn in current recommendations, our results recommended the immediate involvement of high flow oxygen uptake (HFNC) and blood purification treatment via CVVH/CVVHDF at the advanced phase of severe or critical COVID-19 during the standardized treatment. In details, the aforementioned clinical symptoms and laboratory parameters were largely ameliorated, and in particular, the respiratory deficiency (e.g., OC, OI), proinflammatory factors (e.g., IL-6, IL-10) and the accompanying complications (e.g., AKI, ALI/ARDS). For instance, the abnormally upregulated IL-6 and IL-10 with molecular weights ranging from 10 to 30 KD in all cases as previously reported (24) was efficiently eliminated by the filtration membrane (*p* < 0.05), which was in line with Lee's artificial liver therapy in rationales upon cytokine storm (3). Our findings were consistent with the recommendation by Dennis and the colleagues in initiating blood purification therapy during the transition of infection and inflammation from mild to the severe and critical with IL-6 increase for over three times (12, 13). Simultaneously, current studies have suggested the importance of comorbidities and the level of oxygenation at the time of admission, which are fundamental to correlate the effectiveness of new therapies or the implementation of others (13).

However, to our knowledge, the potential influence and direct evidence of continuous blood purification upon COVID-19-assoicated comorbidities and the level of oxygenation at the time of admission are largely unknown (13). Herein, based on the detailed information and preliminary outcomes of the interventional study, we found that continuous blood purification was safe and effective for the remission of the patients, which would provide overwhelming new references for the administration of COVID-19-induced pneumonia. Additionally, BNP is the product of synthesis and secretion of ventricular granulosa cells. When volume overload is associated with heart failure, BNP is significantly increased. As to patients with COVID-19 infection and AKI, the excretion function of the kidney and the metabolic pathway of BNP were impaired, and the increase of BNP was more significant. With the aid of blood purification, the excess fluid could be efficiently eliminated to achieve negative volume balance. Consistently, current studies have also indicated the application of the absorbing filter Oxiris in moderate or severe or critically-ill COVID-19 patients with acute respiratory distress syndrome (ARDS), CSS or secondary infections (26–29).

Increasing number of studies reported that blood purification, such as continuous hemofiltration, hemodiafiltration, hemodialysis, hemoabsorption, plasma exchange, was an effective strategy in controlling the inflammatory cytokine storm (25, 30). For example, Nassiri *et al*. (16) conducted a retrospective study to investigate the effects of extracorporeal hemoadsorption therapy in COVID-19 patients with hyperinflammation and moderate ARDS. They showed that hemoadsorption treatment significantly decreased inflammatory marker plasma concentrations, improved PaO2/FiO2 and organ functions. A case-control, multicenter, prospective study found that blood purification treatment could inhibit the cytokine storm by clearing inflammatory mediators, thus preventing the progress of severe COVID-19 patients and markedly reducing the short-term mortality (31). The national guidelines by the Chinese National Health Commission blood also recommended blood purification for COVID-19 patients with hyperinflammatory response. Consistent with previous studies, this present study showed that the excess inflammatory cytokines were significantly inhibited and the clinical outcomes were improved after blood purification treatment via CVVH/CVVHDF in severe or critical COVID-19. Additionally, radiology examinations of the lung manifestations in patients with COVID-19 infection were not done immediately after every blood purification due to the limitations including transit risk, transit management, isolation and elimination along the way. Instead, the patients were turned to imaging examination after the second or third time of blood purification. Therefore, in this study, we just retrospectively analyzed the data of patients with CVVH/ CVVHDF treatment instead of conducting the comparison with those without blood purification (non-BP treated patients) administration. However, it's interesting to further explore the association between BP and lung manifestations or compare with the control images of non-BP treated patients in future.

Therefore, our study added some evidence for the beneficial effects of early blood purification in severe or critical COVID-19. Meanwhile, considering the single-center observational and comparative study, and the conclusions still require further large-scale confirmation. Besides, it's noteworthy that the potential distinctions among patients with COVID-19-induced pneumonia caused by sexes and ages as well as comorbidities are of equal importance, which are unprocurable for further statistical analysis due to the limitation of sample size in this study. Therefore, prospective, multicenter randomized controlled studies are needed to determine the definitive role of blood purification in controlling inflammatory cytokine storm as well as the mortality in severe and critical COVID-19 patients.

Nevertheless, it's of great importance for clinicians to carry out comprehensive treatment options, including clinical grade vaccines, antiviral drugs (e.g., lopinavir/ritonavir, ribavirin and abidor), antibiotics, cytotherapy (e.g., mesenchymal stem/stromal cells, natural killer cells), anti-inflammatory corticosteroids, immunotherapeutics, Chinese medicine and supportive therapeutics including blood purification (9, 32–35). Overall, on the basis of the retrospective study, we recommended the offhanded implement of CVVH/CVVHDF in case of the occurrence of deteriorated pulmonary ventilation and ground-glass shadows in the lungs to strive for optimal opportunity for treatment as well as alleviate the malignant progression of severe and critical COVID-19 dispense with the long-lasting wait for cytokine examination. Meanwhile, despite the challenge in conducting blood purification, it is feasible to achieve zero infection among medical staffs with the aid of protective measures in the chamber of negative pressure.

## Data Availability Statement

The original contributions presented in the study are included in the article/Supplementary Material, further inquiries can be directed to the corresponding authors.

## Ethics Statement

The studies involving human participants were reviewed and approved by Ethical Committee of Affiliated Hospital of Zunyi Medical University in China (approval number: KLL-2020-013). The patients/participants provided their written informed consent to participate in this study.

## Author Contributions

All authors listed have made a substantial, direct and intellectual contribution to the work, and approved it for publication.

## Funding

The work was supported by Science and technology projects of Guizhou Province (QKH-J-ZK[2021]-107), Natural Science Foundation of Shandong Province (2020QC097), the National Science and Technology Major Projects of China for Major New Drugs Innovation and Development (2014ZX09508002-003), Major Program of the National Natural Science Foundation of China (81330015), China Postdoctoral Science Foundation Project (2019M661033), Natural Science Foundation of Tianjin (19JCQNJC12500), Key Project from Department of Science and Technology of Shangrao City (2020XGFY05), Novel Research & Development Institutions of Shangrao City (2020AB002), and Jiangxi Key New Product Incubation Program from Technical Innovation Guidance Program of Shangrao city (2020G002).

## Conflict of Interest

LZ was employed by the following companies: Guizhou Health-Biotech Biotechnology Co., Ltd., Health-Biotech (Tianjin) Stem Cell Research Institute Co., Ltd., and Jiangxi Health-Biotech Stem Cell Technology Co., Ltd. The remaining authors declare that the research was conducted in the absence of any commercial or financial relationships that could be construed as a potential conflict of interest.

## Publisher's Note

All claims expressed in this article are solely those of the authors and do not necessarily represent those of their affiliated organizations, or those of the publisher, the editors and the reviewers. Any product that may be evaluated in this article, or claim that may be made by its manufacturer, is not guaranteed or endorsed by the publisher.
