# Supplementary material for: Blood Purification in Severe and Critical COVID-19 Patients: A Case Series of 5 Patients
Source: Front Public Health. 2021 Nov 17;9:741125. doi: 10.3389/fpubh.2021.741125 (PMC8635486; doi:10.3389/fpubh.2021.741125)
Supplement: Supplementary file 1 [file Data_Sheet_1.docx]

**Supplementary Information**

**Full title:** Blood purification in severe and critical COVID-19 patients: a case series of 5 patients

**Running title:** Blood purification for COVID-19

Hongjun Chen,^1,#^ Leisheng Zhang,^2,3,4,5, #^ Wei Zhang,^1^ Lili Liu,^6^ Zhihua Dai,^3^ Miao Chen^1, *^ and Donghang Zhang ^7,*^

^1^ Department of Intensive Care Unit, Affiliated Hospital of Zunyi Medical University, Zunyi, 563003, China

^2^ Stem Cell Bank of Guizhou Province, Guizhou Health-Biotech Biotechnology Co., Ltd., Guiyang, 550000, China

^3^ Precision Medicine Division, Health-Biotech (Tianjin) Stem Cell Research Institute Co., Ltd., Tianjin, 301700, China

^4^ Department of Neurosurgery, The First Affiliated Hospital of Shandong First Medical University, Ji-nan, 250014, China

^5^ Jiangxi Research Center of Stem Cell Engineering, Jiangxi Health-Biotech Stem Cell Technology Co., Ltd., Shangrao, 334000, China

^6^ Department of Cerebrovascular Surgery, Affiliated Hospital of Zunyi Medical University, Zunyi, 563003, China

^7^ Department of Anesthesiology, West China Hospital of Sichuan University, Chengdu, China

# These authors contributed equally to this work.

^*^ Corresponding authors.

**Supplementary Information:**

**Supplementary Figures**

**Supplementary Table S1**

**Supplementary Figures**

**
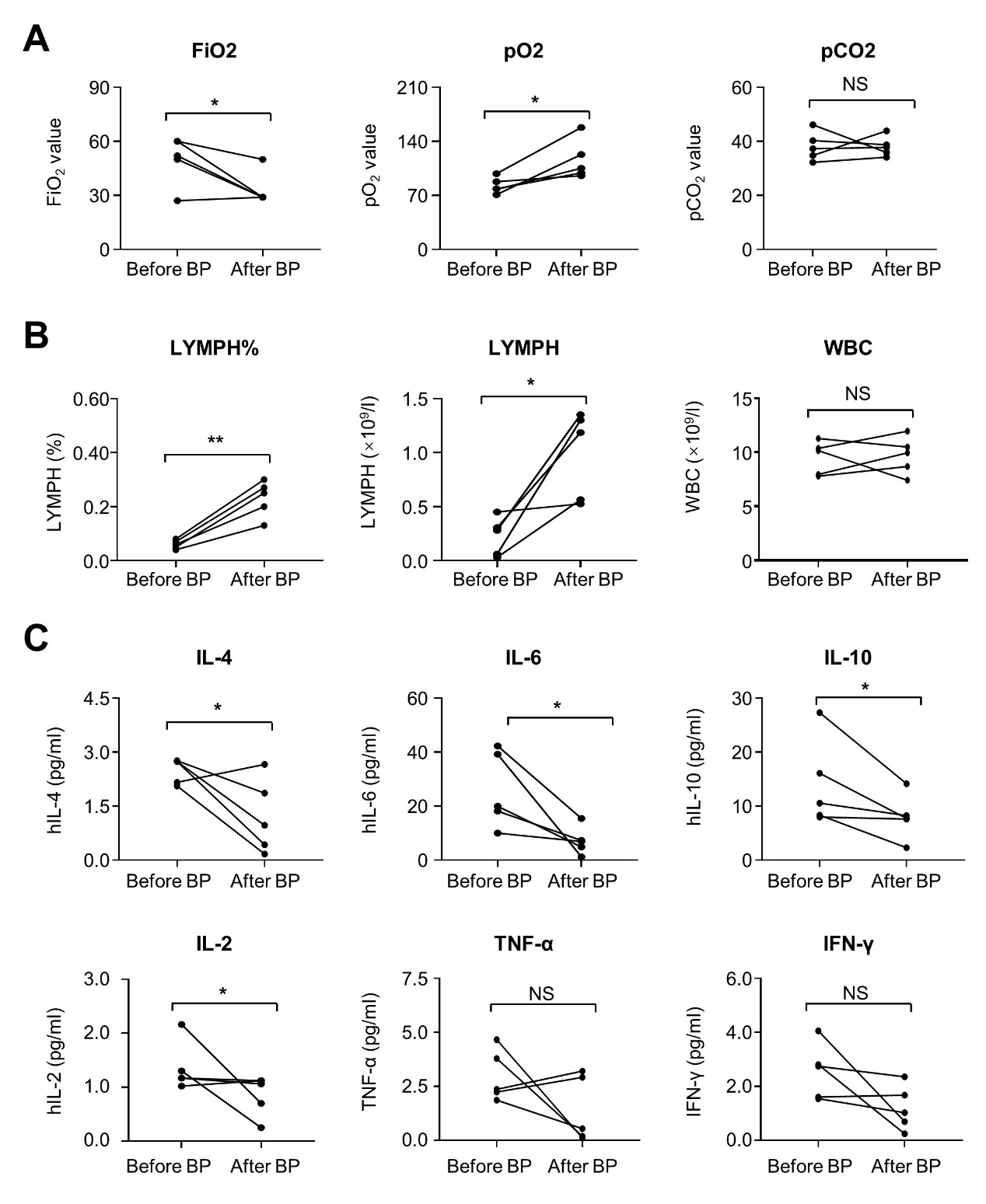
**

**Figure S1. The individual variations of clinical parameters before and after blood purification**

(A) The individual variations of FiO_2_ value, PO_2_ value, PCO_2_ value in the patients before and after blood purification treatment. (B) The individual variations of LYMPH (%), LYMPH and WBC (10^9^/L) in the peripheral blood of the patients before and after blood purification treatment. (C) The individual variations of proinflammatory cytokines (IL-2, IL-4, IL-6, IL-10, TNF-α, IFN-γ) in the peripheral blood of the patients before and after blood purification treatment.

**
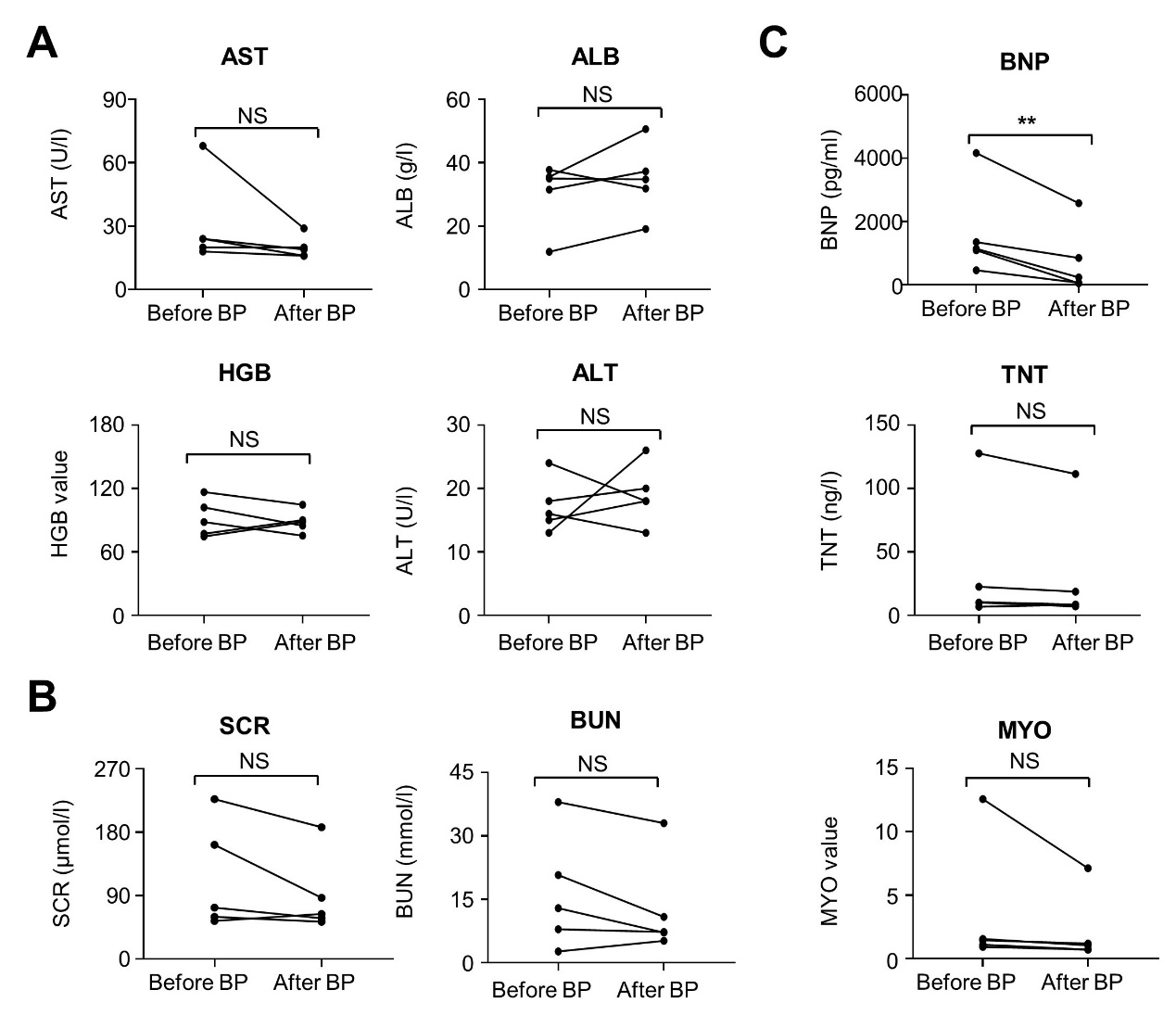
**

**Figure S2. The individual variations of biochemical parameters before and after blood purification**

(A) The individual variations of AST, ALB, HGB and ALT in the patients before and after blood purification treatment. (E) The individual variations of SCR and BUN in the patients before and after blood purification treatment. (F) The individual variations of BNP, TNT, MYO in the patients before and after blood purification treatment. The data were shown as violin diagrams. All the data were shown as Mean±SEM (N=5 individuals). *, P<0.05; **, P<0.01; NS, not significant.”

**Supplementary Table S1**

**Table S1 The choice of CRRT pattern on solute scavenging**

| Pattern | Convection | Dispersion | substitution fluid | dialysis solution |
| --- | --- | --- | --- | --- |
| SCUF | + | - | 0 | 0 |
| CVVH | +++ | - | ++++ | 0 |
| CVVHD | + | +++ | 0 | ++++ |
| CVVHDF | ++ | ++ | ++ | ++ |
